# A New Pro-197-Ile Mutation in *Amaranthus palmeri* Associated with Acetolactate Synthase-Inhibiting Herbicide Resistance

**DOI:** 10.3390/plants14040525

**Published:** 2025-02-08

**Authors:** Meijing Ji, Haiyan Yu, Hailan Cui, Jingchao Chen, Jialin Yu, Xiangju Li

**Affiliations:** 1 State Key Laboratory for Biology of Plant Diseases and Insert Pests, Institute of Plant Protection, Chinese Academy of Agricultural Sciences, Beijing 100193, China; 2 Institute of Advanced Agricultural Sciences, Peking University, Weifang 261322, China

**Keywords:** *Amaranthus palmeri*, resistance, imazethapyr, *ALS* gene mutation, *ALS* gene copy number, *ALS* gene expression

## Abstract

Palmer amaranth (*Amaranthus palmeri* S. Watson), native to North America, is one of the most prominent invasive weed species on agricultural land. Acetolactate synthase (ALS)-resistant *A. palmeri* (*Amaranthus palmeri*) is widespread, while the research focus on resistance pattern and molecular basis of *A. palmeri* to imazethapyr is seldom documented in China. An *A. palmeri* population that survived the recommended rate of imazethapyr was collected in Shandong Province, China. The resistant mechanism and pattern of *A. palmeri* to imazethapyr was investigated. Dose–response assay showed that the resistant (R) population displayed a high resistance level (292.5-fold) to imazethapyr compared with the susceptible (S) population. Sequence analysis of the *ALS* gene revealed that nucleotide mutations resulted in three resistance-conferring amino acid substitutions, Pro-197-Ile, Trp-574-Leu, and Ser-653-Asp, in the individual plants of the R population. An in vitro enzyme assay indicated that the ALS was relatively unsusceptible to imazethapyr in the R population, showing a resistance index of 88.6-fold. *ALS* gene expression and copy number did not confer resistance to imazethapyr in the R population. Pro-197-Ile is the first reported amino acid substitution conferring ALS resistance to *A. palmeri*. This is the first case of an imazethapyr-resistant *A. palmeri* biotype in China.

## 1. Introduction

Palmer amaranth (*Amaranthus palmeri* S. Watson), an annual broadleaf weed species native to the area encompassing the Western United States to Northern Mexico, started to spread beyond its original habitat in the early 20th century [1]. Nowadays, it has infested many crop fields and caused significant yield losses. Yield reduction was up to 68% in soybean [*Glycine Max* (L.) Merrill] fields due to *A. palmeri* (*Amaranthus palmeri*) interference at a density of 10 plants m^−1^ of row [2]. Similarly, researchers reported a reduction of more than 22% in cotton (*Gossypium herbaceum* L.) yield and 11 to 91% in corn (*Zea mays* L.) yield from *A. palmeri* competition at the density of 0.9 plants m^−1^ of row [3,4] and 0.5 to 8 plants m^−1^ of row [5,6], respectively.

Li and Che initially observed *A. palmeri* in Changping District, Beijing, in 1985 [7,8]. At present, this invasive weed has spread to over ten provinces across China [9,10,11]. Due to the environmental adaption and strong competitive capacity, it can grow in cultivated fields, wasteland, ditches, garbage dumps, and feedlots [7]. Recently, an *A. palmeri* population has colonized crop fields and orchards in China [12].

A wide range of pre- and post-emergence herbicides are available for *A. palmeri* control. Among them, acetolactate synthase (ALS)-inhibiting herbicides are used worldwide due to their low cost and low mammalian toxicity. The ALS inhibitors are classified into five chemical families, including sulfonylureas (SUs), imidazolinones (IMIs), pyrimidinylthiobenzoates (PTBs), triazolopyrimidines (TPs), and sulfonylaminocarbonyltriazolinones (SACTs) [13,14,15,16,17]. IMIs, such as imazethapyr, imazaquin, imazapic, and imazameth, are traditionally used for weed control in legumes and non-cultivated land. The long-term use of these herbicides has resulted in the evolution of ALS-resistant populations of *A. palmeri*. Since the first case of imazethapyr resistance in *A. palmeri* in the United States [18], seventeen cases have been reported to be resistant to IMI herbicides worldwide [19].

Currently, target-site and non-target-site resistance are the reported resistance mechanisms to ALS-inhibiting herbicides. However, target-site resistance is the predominant mechanism conferring resistance to ALS-inhibiting herbicides [20]. Generally, target-site resistance is associated with one of several amino acid substitutions in *ALS*. To date, ten amino acid substitutions (Ala-122-Ser, Ala-122-Thr, Pro-197-ser, Pro-197-Ala, Pro-197-Thr, Pro-197-Asp, Ala-282-Asp, Asp-376-Glu, Trp-574-Leu, and Ser-653-Asn) at six positions in *ALS* confer resistance to ALS-inhibiting herbicides in *A. palmeri* globally [21,22,23,24,25], including the cases in Argentina, Brazil, Spain, the United States, Turkey, Italy, and South Africa [26,27,28,29,30,31,32]. In addition, target gene overexpression and gene copy number amplification have also caused the resistance of *A. palmeri* to the herbicides in other groups [33,34]. For example, the copy number and the expression level of 5-enolpyruvylshikimate-3-phosphate synthase (*EPSPS*) genes were correlated with the resistance of a population of *A. palmeri* to glyphosate in Georgia in the United States [35]. Nevertheless, it is still unknown whether copy number amplification and duplication of target genes contribute to some cases of ALS herbicide resistance in *A. palmeri* [21].

In China, there are less relevant reports on herbicide-resistant *A. palmeri.* In 2020, Xu et al. collected some populations of *A. palmeri* conferring resistance to ALS herbicide from Beijing, Hebei, Guangxi, Jiangsu, and Fujian; however, their resistance level and mechanism are still unknown [11]. In 2019, a population of *A. palmeri* in Shandong, China, survived following imazethapyr applications at the recommended rate in our yearly survey. The objective of this research was to characterize the level of imazethapyr resistance in this *A. palmeri* population and to identify the biochemical and molecular bases of the herbicide resistance.

## 2. Results

### 2.1. Response of Amaranthus palmeri to Imazethapyr

**Figure 1:**
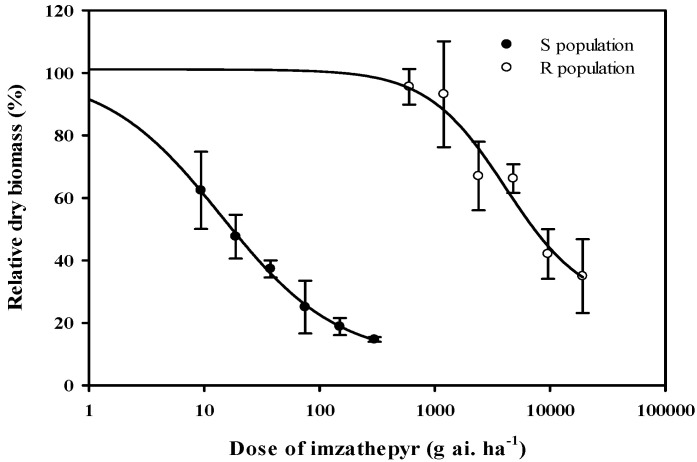
Dry shoot mass reduction in the susceptible (S) and the resistant *Amaranthus palmeri* population (R) at 21 d after treatment with imazethapyr in two growth chamber experiments. Results were pooled over experimental runs. Vertical bars represent the standard error of the mean (n = 6).

Increasing imazethapyr doses caused shoot mass reduction compared to the nontreated control for both R and S populations. However, the S population of *A. palmeri* exhibited substantially greater susceptibility to imazethapyr than the R population, as shown in Figure 1. From the regression analysis, GR_50_ values were 4124.6 g ai. ha^−1^ and 14.1 g ai. ha^−1^ for the R and S populations, respectively, indicating that the R population had a 292.5-fold higher resistance to imazethapyr than the S population. Therefore, these findings suggested that the R population of *A. palmeri* exhibited a high level of resistance to imazethapyr.

### 2.2. Sequencing of ALS Gene

**Figure 2:**
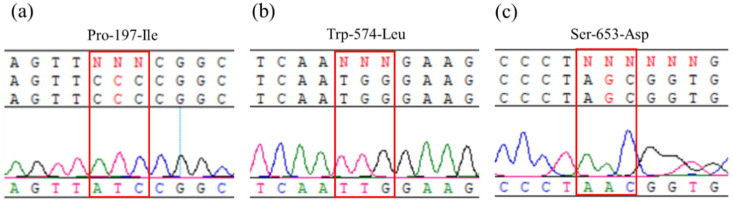
Target-site mutations in the *ALS* gene conferring herbicide resistance in the R population: (**a**) Pro-197-Ile (CCC mutated to ATC), (**b**) Trp-574-Leu (TGG mutated to TTG), and (**c**) Ser-653-Asp (AGC mutated to AAC) in the *ALS* gene sequence.

*ALS* gene sequencing from 100 individual plants of the R population in *A. palmeri* revealed that the population possessed three different known *ALS* resistance mutations, tryptophane (TGG) to leucine (TTG) at position 574 (Trp-574-Leu), serine (AGC) to aspartic acid (AAC) at position 653 (Ser-653-Asp), and proline (CCC) to isoleucine (ATC) at position 197 (Pro-197-lle) (Figure 2). No other known *ALS* resistance mutations were found. Nevertheless, it is worth mentioning that Pro-197-lle mutation had not been previously reported in *A. palmeri*.

Of the 100 analyzed individual plants from the R population, 19 plants had Pro-197-Ile mutation, and 20 individuals had Ser-653-Asp mutation, whereas only 13 plants carried the Trp-574-Leu mutation. Additionally, many individuals displayed more than one *ALS* gene mutation, with 45% of individuals having two mutations (197 + 574, 197 + 653, 574 + 653), two plants with all three resistance mutations (197 + 574 + 653), and one plant did not contain any of the known *ALS* gene mutations. Trp-574-Leu and Ser-653-Asp mutations were more common than Pro-197-lle mutations (Table 1).

### 2.3. Results of ALS Enzyme Activity Test In Vitro

**Figure 3:**
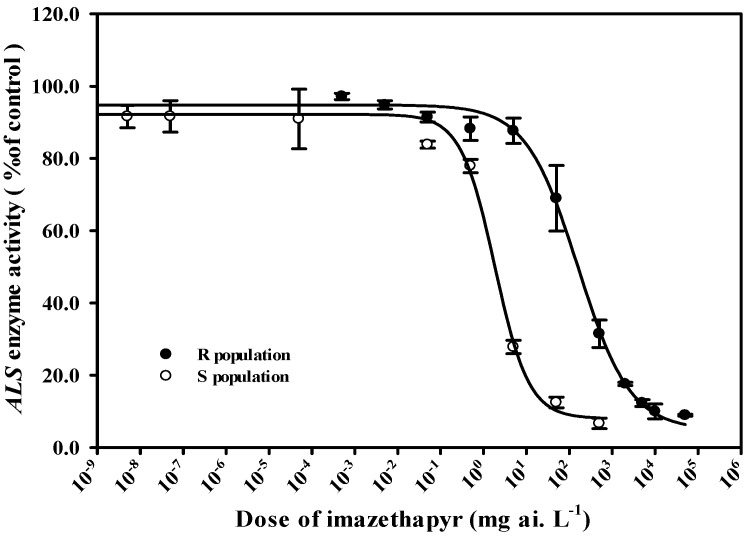
In vitro ALS activity assays in response to imazethapyr were performed using protein extracts of plants derived from the resistant *A. palmeri* (*Amaranthus palmeri*) population (●) and the susceptible population (○). Results were pooled over experimental runs. Vertical bars represent the standard error of the mean (n = 3).

As the herbicide dose increased, the ALS enzyme activity relative to the nontreated control decreased for both S and R populations in *A. palmeri* (Figure 3). Nevertheless, the ALS enzyme activity of the S population sharply declined and showed substantially greater sensitivity to imazethapyr than the R population. The IC_50_ value of the S population was 1.8 mg ai. L^−1^, while the IC_50_ value of the R population was 159.5 mg ai. L^−1^ when calculated based on acetoin (acetolactate) produced per mg of fresh weight. The IC_50_ of the R population was 88.6-fold greater than that of the S population. Thus, the result suggested that the resistance to imazethapyr in the R population is associated with the target-site resistance mechanism.

### 2.4. ALS Gene Copy Number and ALS Gene Expression

**Figure 4:**
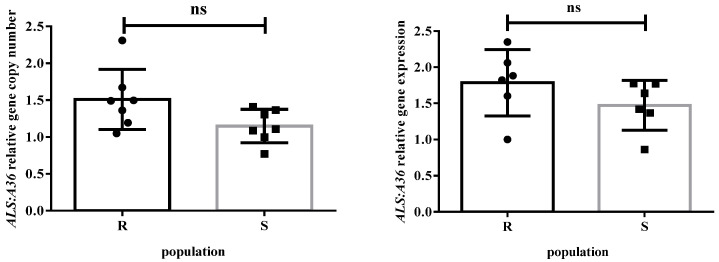
*ALS* copy number relative to *A36* for the S and R populations; *ALS* gene expression relative to *A36* for the S and R populations not treated with imazethapyr. Vertical bars represent the mean ± standard deviation; ns represents non-significant.

There were no significant differences in the *ALS* gene copy numbers between the S and R populations. The *ALS* gene copy number relative to internal reference gene *A36* for both S and R populations ranged from 1.0 to 2.3 (Figure 4). Similarly, the *ALS* gene expression levels in the R population did not significantly differ from the S population. Without imazethapyr treatment, the *ALS* gene expression level in the R population ranged from 1.6 to 2.3, while the level in the S population ranged from 0.9 to 1.8 (Figure 4). These findings indicated that the *ALS* gene copy numbers and gene expression levels are unlikely to be associated with the resistance in the R population.

## 3. Discussion

*Amaranthus* species have the most cases of resistance to various herbicide modes of action because of their high genetic diversity, high seed production, and continuous emergence [36,37]. For example, a prostrate pigweed (*Amaranthus blitoides* S. Watson) population demonstrated 790-fold resistance to ALS inhibitors [38], and a smooth pigweed (*Amaranthus hybridus* L.) population exhibited up to 537-fold resistance to imazethapyr [39]. An early investigation conducted in the United States showed that resistant *A. palmeri* biotypes had greater than 2800-fold resistance to imazethapyr than the susceptible biotypes [40]. In the present research, the R population displayed resistance to imazethapyr (292.5-fold) at the whole plant level, and the IC_50_ value at the enzyme level of the R population was 88.6-fold greater than that of the S population. These findings suggested the R population of *A. palmeri* investigated in the present study has evolved a high level of resistance to imazethapyr.

In this study, *ALS* gene sequencing identified three amino acid substitutions, including Pro197, Trp574, and Ser653, in the R population. These mutations agree with the previous reports in *A. palmeri* populations [19]. However, it should be noted that Pro-197-lle mutation has not been previously reported in *A. palmeri* in the literature. To date, the substitution (Pro-197-Ile) has been found in *Amaranthus arenicola* and *Sisymbrium orientale* L. [11,19]. Importantly, many individual plants of the R population in the present study displayed more than one *ALS* gene mutation, with 45% of individuals possessing two resistance mutations (197 + 574, 197 + 653 and 574 + 653) and 2% of the plants carrying all three mutations (197 + 574 + 653). Multiple gene mutations may be related to the fact that *A. palmeri* is an obligate outcrossing, dioecious species. A plant with no mutation detected in the *ALS* gene sequencing may be due to the accumulation of a pair of recessive genes during the reproduction of the population.

It is worth mentioning that a single *ALS* gene has two or more *ALS* mutations (double or multiple mutations) at the allele level, and this may complicate the resistance to ALS-inhibiting herbicides situation [20]. However, the epistatic (multiplicative, additive, synergistic, and compensatory) effect of multiple resistance alleles on plant fitness cost is not yet clear when individual plants accumulate multiple *ALS* resistance alleles [20]. In the present study, the proportion of double or triple mutations in all plants tested was nearly 50%. Thus, an additional study should be conducted to confirm the contribution of multiple *ALS* resistance alleles in different mutated biotypes in the R population for the resistance to imazethapyr.

Target gene amplification and overexpression have contributed to the herbicide resistance in some weed species [34,35,41]. In glyphosate-resistant plants, including *Amaranthus* spp., *EPSPS* gene amplification is the primary resistance mechanism [35,42]). In the R population of goosegrass (*Elusine indica* L.), the expression of *EPSPS* was higher (13.8-fold) than that of the S population after glyphosate treatment [34]. *Amaranthus* spp. is a diploid plant that contains one copy of the *ALS* gene [43]. However, the intense selection pressure of ALS inhibitors may promote the genotypes with increased *ALS* gene copies, resulting in herbicide resistance [21]. In the present study, the *ALS* gene copy numbers were not significantly different between the R and S populations. Without imazethapyr treatment, the *ALS* gene expression level in the R and S populations did not exhibit a significant difference. These findings suggested that the slight change (elevation or depression) in relative *ALS* gene copy number and gene expression in this ALS-resistant *A. palmeri* population did not contribute to the resistance.

As an invasive weed in China, the development of herbicide-resistant *A. palmeri* will undoubtedly affect the ecological environment and agricultural production. In the United States, crop losses from invasive weeds have been estimated at approximately USD 27,000 million per year [44]. The development of high level of resistance in *A. palmeri* will bring further risks to crop production. Multiple hypotheses may explain the resistance development observed in this population. Peanuts, soybeans, and other crops are widely cultivated in the local area, and imazethapyr is commonly used for weed control in these crops. The long-term use of this herbicide may have led to the development of resistance. Alternatively, cross-resistance patterns may have developed through prolonged exposure to other ALS-inhibiting herbicides with similar modes of action. Another possibility is that the invasive species itself carried resistance genes. Additionally, the collection site is near an herbicide manufacturing plant that produces imazethapyr, which could have induced herbicide resistance in the weeds.

## 4. Materials and Methods

### 4.1. Plant Materials and Growth Conditions

Matured *A. palmeri* seeds of a suspected resistant (R) population were collected on a roadside near a ditch in Shandong, China, while a susceptible (S) population were collected in a corn field in Beijing, China. The collected seeds were propagated for one year under controlled conditions. The seeds of the R and S populations were planted in plastic pots (10 cm in diameter and 10 cm in height) containing commercial potting soil (50% peat, 25% pine bark, and 25% sand) (Beijing Kawin Technology Share-Holding Co., Ltd., Beijing, China) and placed in a greenhouse at 30/25 °C day/night with natural sunlight. The plant seedlings were watered as needed. The seedlings were thinned to 5 plants per pot when they reached a 2- to 3-leaf stage.

### 4.2. Dose–Response Experiments

To determine the resistance level of the R population to imazethapyr, experiments were conducted in August and December 2020. Plants at the 4- to 6-leaf stage were treated with imazethapyr (Doushile^®^, 5% AS, Shandong Cynda Chemical Co., Ltd., Shanghai, China) in a spray cabinet (3WPSH-500D, Beijing Research Center for Information Technology in Agriculture, Beijing, China) equipped with a single moving Teejet XR 8003 flat fan nozzle and calibrated to deliver 367.5 L ha^−1^. Imazethapyr rates applied to the S population were based on 0, 0.125×, 0.25×, 0.5×, 1×, 2×, and 4× the recommended rate, while imazethapyr rates applied to R populations were based on 1×, 2×, 4×, 8×, 16×, 32×, 64×, 128×, and 256× the recommended rate (recommended field rate = 75 g ai. ha^−1^). Shoots were harvested at 21 days after treatment (DAT), oven-dried at 80 °C for 72 h, and then weighed.

Experiments were designed as a randomized complete block with three replications and were conducted twice over time. Dose–response curves were generated by regression analysis using SigmaPlot v. 13 (Systat Software, Inc., San Jose, CA, USA). Data were regressed with a three-parameter log-logistic equation (Equation (1)):

(1) $$Y=C+\frac{D-C}{1+{\left(X/{GR}_{50}\right)}^{b}}$$

where Y represents the shoot dry weight (percentage of nontreated control) at herbicide rate X, C is the lower limit, and D is the upper limit. Imazethapyr rate that caused 50% shoot mass reduction (GR_50_) was determined from the regression equation.

### 4.3. ALS Gene Sequencing and Resistance Mutation Genotyping

Genomic DNA was extracted from young leaf tissues of 100 nontreated plants in the R population, in which seeds came from the reproduction of plants that survived the imazethapyr treatment in the previous study. Genomic DNA extraction was conducted according to the kit instruction (Tiangen Biotech Beijing Co., Ltd., Beijing, China). Two primer pairs were designed based on the plant *ALS* gene sequence (KY781923.1) of *A. palmeri* and amplified the region containing the eight mutation sites that have been reported in other resistant plant species (Table 2). Three mutation sites (574, 653, and 654) were amplified with the primers (*ALS*-1199f/*ALS*-1199r: TGCCTAAACCCACTTATTCTGC; ATCTCCAACCAACTAATAAGCC). The sequence was amplified by the other primers (*ALS*-921f/*ALS*-921r: TTTGTTTCCCGATTTAGTCCT; AACAAATCGGCCTTATCAACC) containing five mutation sites (122, 197, 205, 376, and 377).

The PCR reaction consisted of 12.5 μL 2× Pfu PCR Master Mix (Tiangen Biotech Beijing Co., Ltd., China), 10.5 μL ddH_2_O, 0.5 μL the forward and reverse primers (Tiangen Biotech Beijing Co., Ltd., China), and 1 μL gDNA to make a 25 μL total volume. PCR was performed with the following conditions: initial denaturation at 95 °C for 3 min, followed by 35 cycles of denaturation at 94 °C for 30 s, annealing at 55/53 °C for 30 s, and extension at 72 °C for 1 min, and a final extension at 72 °C for 10 min. The PCR tubes were held at 4 °C until processing. PCR products were visualized on a 1% agarose gel to confirm the target fragment size. The PCR products were sequenced commercially (Beijing Sunbiotech Co., Ltd., Beijing, China), and the sequencing results were analyzed using DNAMAN version 5.2.2 software (Lynnon Biosoft, Quebec, QC, Canada).

### 4.4. In Vitro ALS Assay

Leaf materials at the 4- to 6-leaf stage were harvested. All samples were stored at −80 °C until use. ALS enzyme extraction and herbicide inhibition assays were conducted according to Yu [45]. A series of imazethapyr concentrations (0, 5 × 10^−4^, 5 × 10^−3^, 5 × 10^−2^, 0.5, 5, 50, 500, 2000, 5000, 10,000, 25,000, and 50,000 mg ai. L^−1^ for the R population; 0, 5 × 10^−8^, 5 × 10^−7^, 5 × 10^−6^, 5 × 10^−5^, 5 × 10^−4^, 5 × 10^−3^, 5 × 10^−2^, 0.5, 5, 50, and 500 mg ai. L^−1^ for the S population) was used. The ALS enzyme activity assay was conducted twice over time with three replications per treatment. Data were regressed with the equation previously described in Section 2.2.

### 4.5. ALS Gene Copy Numbers and ALS Gene Expression

Leaf tissues for the R and S populations were collected from the nontreated plants, frozen immediately with liquid nitrogen, and stored at −80 °C until processing. Genomic DNA was extracted using the methods previously described in Section 2.3. The primers for the internal reference genes *A36* *A36*_F244 (5′-TTGGAACTGTCAGAGCAACC-3′) and *A36*_R363 (5′-GAACCCACTTCCACCAAAAC-3′) were designed by Singh [46]. For the *ALS* gene, the primers *ALS*-F2 (*5′*-GCAATTCCTCCGCAATACGCC-*3′*) and *ALS*-R2 (*5′*-CAAACCCCATAGCCCCCAAAC-*3′*) were designed with Oligo version 7 software and based on the GenBank entry KY781922.1 for use in quantitative PCR on genomic DNA and cDNA. *ALS* primers were designed based on conserved regions in published plant *ALS* gene sequences. Efficiency curves were made for each primer set using a 0.0625×, 0.125×, 0.25×, 0.5×, and 1× dilution series of genomic DNA from the R population.

The relative *ALS* gene copy numbers were investigated using qPCR. A 25 μL reaction solution was prepared using 10 μL of Bester SybrGreen Master Mix (DBI^®^ Bioscience, Ludwigshafen, Germany), 0.5 μL of forward and reverse primers (Beijing Sunbiotech Co., Ltd., Beijing, China), 8 μL of RNase-free water, and 0.04 μL of 50× ROX Reference Dye (DBI^®^ Bioscience). qPCR was performed with the following steps: initial denaturation at 95 °C for 4 min, followed by 40 cycles of denaturation at 95 °C for 20 s, and annealing at 60 °C for 1 min. This program was followed by a melt curve analysis of 81 cycles of 55 °C for 30 s. The negative control contained no template in the reaction. No amplification products were detected in the negative control. Data were analyzed using a modification of the 2^−ΔΔCt^ method to determine the genomic copy number of *ALS* relative to *A36* as ΔCt = (Ct, *A36* − Ct, *ALS*), and the relative increase in the genomic *ALS* copy number was expressed as 2^ΔCt^. Each reaction had three technical replicates.

The nontreated plant leaf tissues from the R and S populations were collected, immediately frozen with liquid nitrogen, and then stored at −80 °C until processing. Total RNA was extracted using an RNA-prep Pure Plant Kit (Tiangen Biotech Beijing Co., Ltd., Beijing, China). First-strand complementary DNA was synthesized using a FastQuant RT Kit (Tiangen Biotech Beijing Co., Ltd., Beijing, China). Quantitative real-time PCR was performed in a 25 µL reaction on a PCR machine (7500 Fast Real-Time, Thermo Fisher Scientific, Shanghai, China) under the following conditions: 10 min at 95 °C, 40 cycles of 95 °C for 20 s and 60 °C for 1 min, and then increasing the temperature by 0.5 °C every 5 s to obtain the product melt curve. Relative quantification of *ALS* was calculated using the 2^−ΔΔCt^ method and Ct = − [(Ct, *ALS* − Ct, *A36*) R − (Ct, *ALS* − Ct, *A36*) S]. The results are expressed as the fold increase in *ALS* expression level relative to *A36*.

## 5. Conclusions

In summary, this study reported the first case of imazethapyr-resistant *A. palmeri* in China. Various mutations in *ALS* caused a high level of resistance to imazethapyr, and a new amino acid substitution (Pro-197-Ile) was detected in the R population of *A. palmeri*. In dioecious species, resistance genes can be exchanged or transferred between the plants [47]. Cross- or multiple-resistance is common in *A. palmeri* [19,42,48,49]. The case of an *A. palmeri* population with multiple-resistance to 2,4-D, atrazine, chlorsulfuron, glyphosate, and mesotrione was confirmed in Kansas in the United States [50]. Therefore, additional studies need to be conducted to confirm the resistance levels of different mutation types and the cross- and multi-resistance patterns in the R population. Integrated weed management should be implemented to reduce the risk of further genetic evolution and the spread of resistant *ALS* gene in *A. palmeri* in China.

## Figures and Tables

**Table 1:** *ALS* domain sequencing results show one, two, or three different mutations in 100 individual plants of the R population.

Gene Mutation Types	Number of Individual Plants
Pro-197-lle	19
Trp-574-Leu	13
Ser-653-Asp	20
Pro-197-lle+ Trp-574-Leu	11
Pro-197-lle+ Ser-653-Asp	9
Trp-574-Leu+ Ser-653-Asp	25
Pro-197-lle+ Trp-574-Leu+ Ser-653-Asp	2
Mutation not detected	1

Abbreviation: *ALS*, acetolactate synthase.

**Table 2:** Primers for partial *ALS* sequencing of *Amaranthus palmeri.*

Primer	Sequence (5′–3′)	Covered Sites	Annealing Temperature (°C)	Amplicon Size (bp)
*ALS*-1199f	TGCCTAAACCCACTTATTCTGC	574, 653, 654	55	1199
*ALS*-1199r	ATCTCCAACCAACTAATAAGCC			
*ALS*-921f	TTTGTTTCCCGATTTAGTCCT	122, 197	53	921
*ALS*-921r	AACAAATCGGCCTTATCAACC	205, 376, 377		

## Data Availability

The sequence of *A. palmeri ALS* gene has been deposited in the NCBI with GenBank accession numbers of KY781923.1 and KY781922.1.
